# An analysis of fertility and fecundity in the Australian sheep flock between 2006 and 2019

**DOI:** 10.1038/s41598-024-67847-4

**Published:** 2024-08-01

**Authors:** G. Refshauge, M. Golledge, J. Rickard, S. de Graaf

**Affiliations:** 1 New South Wales Department of Primary Industries and Regional Development, Cowra Agricultural Research and Advisory Station, Cowra, NSW 2794 Australia; 2 Faculty of Science, School of Life and Environmental Sciences, The University of Sydney, Sydney, NSW 2006 Australia

**Keywords:** Reproductive biology, Infertility

## Abstract

After decades of decline, the Australian sheep flock aspires to rebuild its population of breeding ewes. A successful, rebuild will rely on high pregnancy rates and number of lambs born and reared. To examine this potential, a cross-sectional study of historical ultrasound pregnancy scanning records was undertaken using records collated from two experienced sheep pregnancy scanning businesses (years 2006 to 2019) from 15,397 mobs of ewes, totalling 7,443,314 ewes. Client details were de-identified and excluded from analyses, but details describing the mobs were retained when available, such as season of mating, production zone, ewe age, and breed. The key finding was a mean pregnancy rate (ewes pregnant per ewe scanned) of 0.76 ± 0.24, with a median of 0.83. Mobs scanned to identify fetal number had a higher mean (0.84 ± 0.15) and median (0.89) pregnancy rate. The mean reproduction rate (fetuses per ewe scanned) was 1.21 ± 0.27 and the median was 1.25. Differences were observed between the factors including age, breed, season, year or production zone but all results were lower than anticipated. The unexpected findings imply a problem exists with the fertility of many Australian sheep flocks.

## Introduction

After decades of decline, the Australia sheep flock aspires to rebuild its population of breeding ewes^1^. A successful, rebuild will rely on reproductive efficiency, retention of older ewes, and the use of real-time ultrasound pregnancy scanning. Real-time ultrasound is a cheap, practical and fast way to determine the pregnancy status of a sheep and can be confidently and accurately performed between gestational ages of 40 to 100 days post-conception^2^. Incorporating ultrasound pregnancy scans into the management operations of sheep farms allows metrics to be collected on the various sources of reproductive wastage^3^. Reproductive loss to mid-pregnancy varies greatly due multiple factors^4^, for example, liveweight^5^ and body condition score^6^ are important factors to consider when preparing ewes for mating. Other important temporal considerations include disease^7^, ewe and ram nutrition^8^, and climatic extremes^9^. Once committed to a breeding program, factors such as breed^10–12^, age^13,14^, season of mating^15^, location^16^ and genetics^17^ become important factors affecting reproduction outcomes that are less easily changed in a short period of time without major planning and re-investment. Understanding how these factors interact to affect ewe fertility and fecundity underscores the improvement of reproductive performance, farm productivity, and industry profitability.

There is, however, currently no robust, comprehensive dataset describing historical ewe reproductive performance and its variability across Australian regions. We proposed that such information existed within pregnancy scanning businesses and if large datasets were collated over protracted timeframes, considerable power could be drawn upon to focus on issues affecting sheep reproductive potential. This paper reports key findings using 14 years of pregnancy scanning data collected from two sheep pregnancy scanning businesses.

## Methods

### Data collation

**Figure 1:**
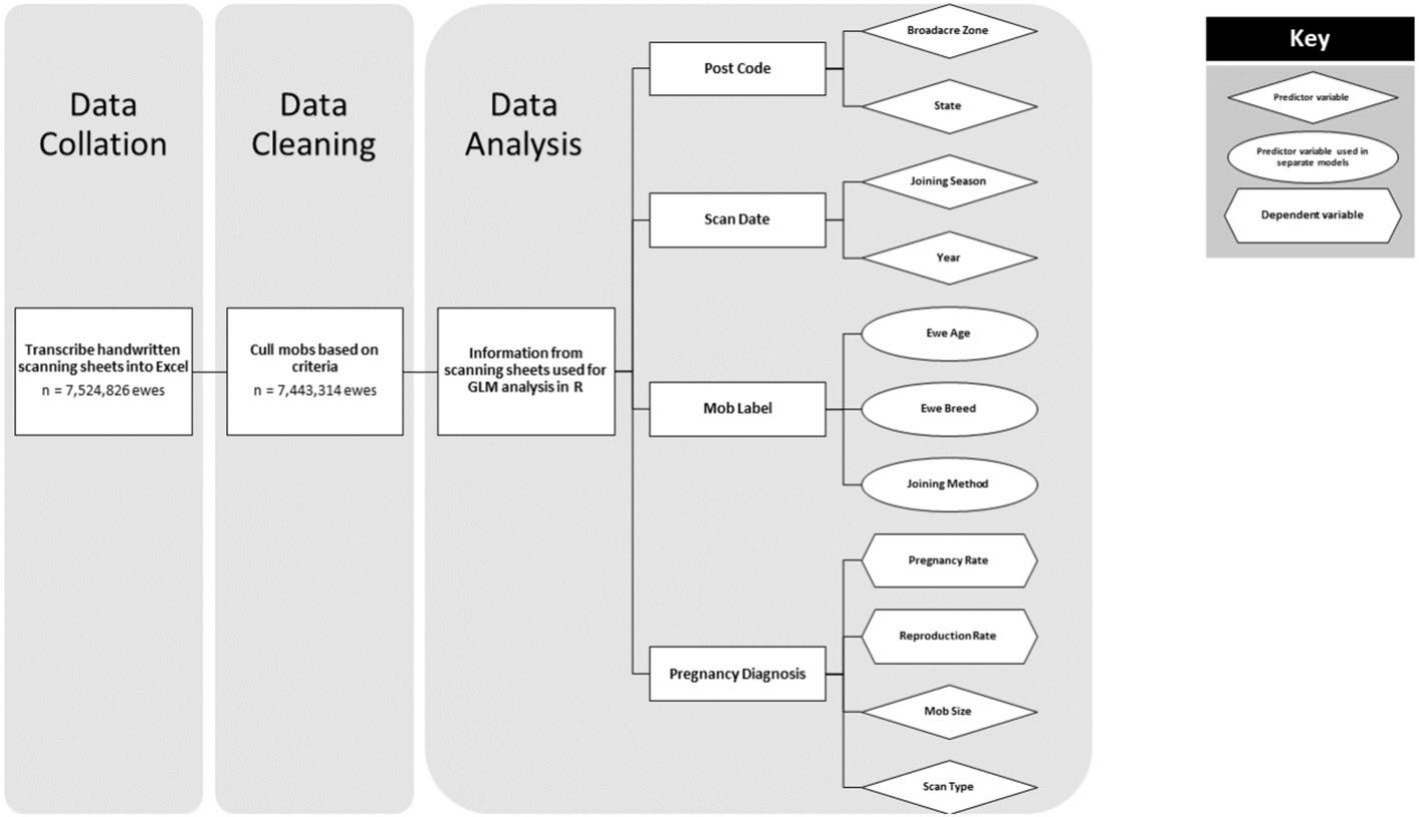
Flowchart describing the process to synthesise the original data through to the factors and variables available for analysis.

Electronic and handwritten pregnancy data sheets from 15,397 mobs across all Australian states and territories (ACT, NSW, NT, QLD, SA, VIC, WA) were collated from two experienced, professional pregnancy scanning contracting businesses. These sheets provided information on 7,524,826 ewes. The data sheets were collected for years covering 2006 through to 2019. Only one business provided data for 2015, due to flood damage destroying the 2015 records at the other business. A flow chart describing data synthesis is provided (Fig. 1).

Pregnancy scanning sheet information per mob were transcribed into Microsoft Excel, including some or all of client name, trading name, town, postcode, scanner’s name, date of scanning, number of empty (non-pregnant) ewes, number of ewes with a single fetus, number of ewes with multiple fetuses (if scanned for litter size), total ewes scanned, and reproduction rate, and when available specific mob labels such as ewe breed, age and in some instances detail indicating whether the ewes were artificially inseminated or were embryo transfer recipients.

### Data cleaning

Mobs were removed from the data set if the total number of sheep scanned per mob recorded on the pregnancy scanning sheet did not match the re-calculated total of sheep per mob based on the sum of ewes with 0, 1, and 2 fetuses. Mobs with trading names of known saleyards or specialist feedlots were not included in the dataset because in those instances scanning date and town name information on the pregnancy scanning sheet would not be an accurate depiction of the time or location of joining of ewes scanned. In addition, it is expected that these mobs were scanned to ensure lack of a fetus prior to market, rather than representing ewe fertility. After cleaning, data prepared for analysis totalled 15,233 mobs with 7,443,314 ewes scanned for pregnancy and 3,073,866 ewes scanned for multiple fetuses from 4,761 mobs. Post codes and town names were then used to identify geographical regions of Australia associated with climate and agriculture production using the Broadacre Zones (High Rainfall Zone, Wheat-sheep Zone, or Pastoral Zone), as defined by ABARES^18^. Table 1 provides the ewe numbers by state and region.

**Table 1:** Ewe numbers for each agricultural production zone and each state or territory.

State or Territory	Pastoral Zone	Wheat-Sheep Zone	High Rainfall Zone	Unknown	Total
Australian capital territory			1,314		1,314
New south wales	279,065	1,680,048	568,612	8,433	2,536,158
Northern territory	4,142			938	5,080
Queensland		4,368	4,754	14,710	23,832
South australia	277,648	1,781,664	501,192	71,139	2,631,643
Tasmania			6,400		6,400
Victoria	1,270	19,096	12,536	4,285	37,187
Western australia	23,075	1,166	1,873	418	26,532
Not available	15,984	8,745	8,654	2,141,785	2,175,168
Total	601,184	3,495,087	1,105,335	2,241,708	7,443,314

To provide client confidentiality, all personal information was de-identified. Town names and post codes were removed from the dataset to exclude specific location of properties. Client and trading names were completely discarded and replaced with numerical client IDs, followed by replacing scanner names with a coded identifier. Therefore, all personal information remains strictly confidential.

Following de-identification, date of scanning was utilized to estimate the joining season and joining year. The start date for joining was estimated by subtracting 86 days from date of scanning. End of joining was determined by adding 42 days to the start of joining. The season of joining was determined by the month of start of joining, with mobs that began joining in December to February labelled as summer, March to May as autumn, June to August as winter, and September to November as spring.

Ewe age information was available for 1,440,115 ewes scanned for pregnancy status and 468,564 scanned for multiple fetuses. Ages were classed into ‘lamb’, ‘maiden’ and ‘mature’ based on available information, such as the name of age group, years old, and/or NLIS coloured tags representing year of birth. The age class for lambs were defined as ewes under 1 year of age; hoggets and maidens as 1–2 years of age; and mature as over 2 years of age. Mob labels that indicated mixed ages were assigned, ‘mature’.

When available, mob labels included information regarding ewe breed were grouped into ‘Maternal’, ‘Merino’, ‘Terminal, and ‘Shedders’. Terminal breeds in this dataset include Dorset, Suffolk, Whiteface, Blackface, and Texel; maternal breeds include Border Leicester, South African Mutton Merino, Corriedale, and crossbreds; shedder breeds include Dorper, Wiltshire Horn, and Ocala Hair; Merino breeds include Dohne and Merino. Ewe breed information was available for 1,794,616 ewes scanned for pregnancy and 610,337 scanned for multiple fetuses.

Pregnancy rate was calculated using the number of pregnant ewes per number of ewes scanned per mob. Reproduction rate was calculated using the number of fetuses identified per number of ewes scanned per mob. Mobs that were not scanned for multiples are described as scanned in lamb ‘(SIL)/Empty’, while mobs scanned for fetal number are described as ‘multiples’.

Mob sizes were based on total sheep scanned per mob and grouped by under 100, 100 to 200, 200 to 500, 500 to 1000, and > 1000 ewes scanned per mob.

### Data analysis

The analysis was performed utilizing Generalized Linear Models (GLMs) with a logistic regression approach to predict the pregnancy rate and reproduction rate of ewes. The models incorporated various predictor variables, such as type of scan (SIL/Empty or multiple), season of mating, and broadacre zone, which were weighted based on the total number of ewes scanned per mob. However, the implementation of a mixed model was not feasible due to multicollinearity issues when attempting to incorporate ewe age, breed, method of mating, and mob size as predictor variables. As a result, separate logistic regression models were fitted for each of these variables.

The analysis was executed using the R programming language^19^. The p-values were estimated using the Wald z-distribution approximation. The magnitude of the effect of each predictor variable was quantified in Microsoft Excel^™^ by examining the exponentiated beta coefficients, which were calculated using the formula 1 + e^(−difference between intercept and the predictor variable)/1 + e^(−intercept). The model outputs and log-likelihood outcomes are provided as Supplementary Material.

## Results

**Figure 2:**
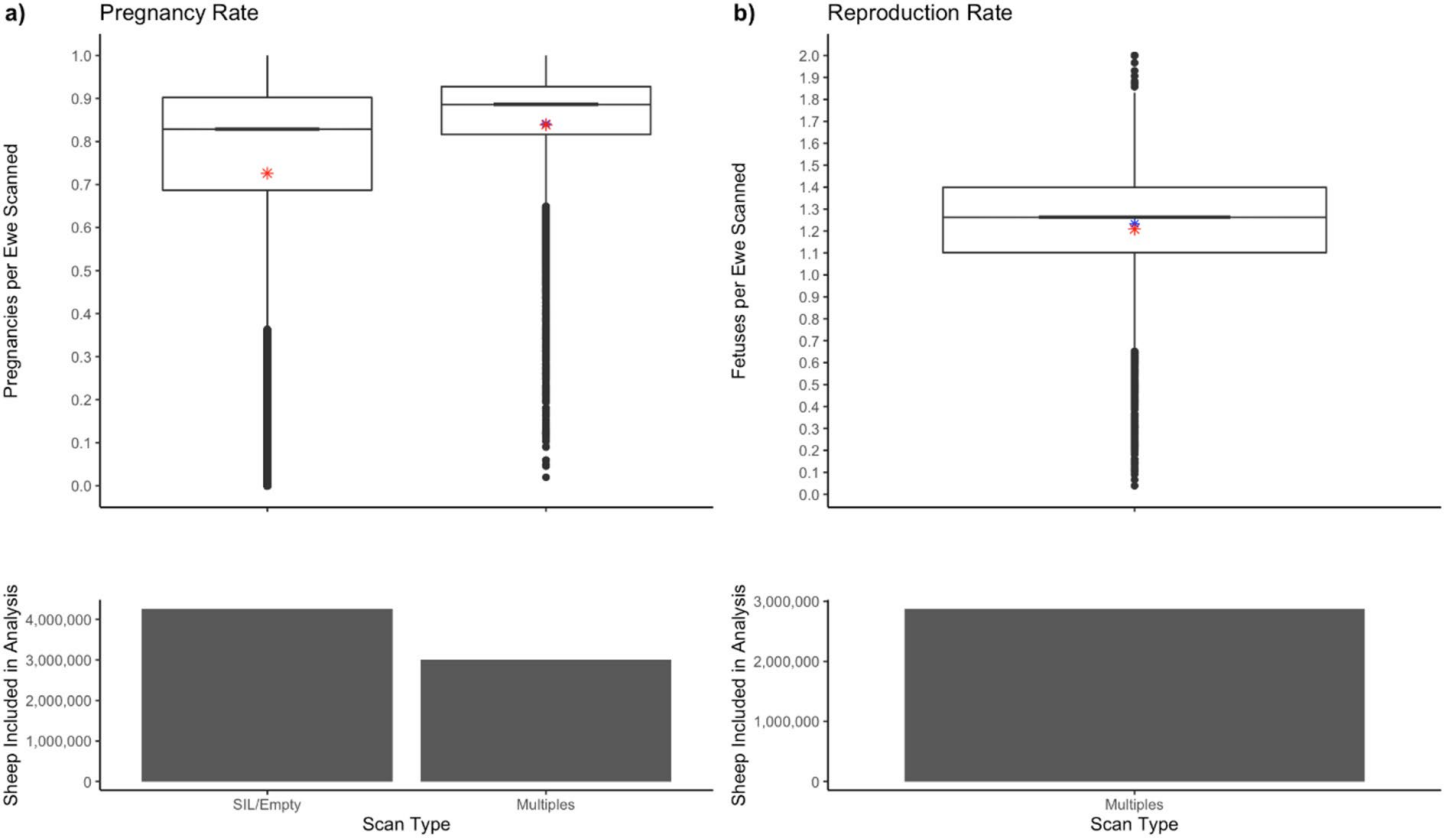
Boxplots for the types of pregnancy scan. The column graphs indicate the number of ewes scanned. Panel (**a**) Pregnancy rate (ewes pregnant per ewe scanned) for ewes scanned to identify pregnancy status only (SIL/Empty) and those scanned for multiple fetuses. Panel (**b**) Reproduction rate (fetuses per ewe scanned). Boxplots indicate the range, average (red star), median (black bar) and predicted mean (blue star).

The average pregnancy rate for all mobs was 0.76 ± 0.24 (S.D.), while the median was 0.86. The average pregnancy rate of mobs scanned for litter size was 0.84 ± 0.15. The mean reproduction rate was 1.21 ± 0.27 with a median of 1.25 fetuses per ewe (Fig. 2). The number of ewes pregnancy scanned in each state and territory are as follows: South Australia 2,631,643, NSW 2,536,158, Victoria 37,187, WA 26,532, QLD 23,832, Tasmania 6,400, NT 5,080, ACT 1,314 and unknown location with no post code recorded 2,175,168.

A full list of exponentiated beta coefficients is available for all models in Supplementary Table 1.

### Type of scan

Only 31% of mobs were scanned for multiples but the average size of the multiple-scanned mobs was 50% higher than mobs scanned empty or in lamb (633 v 423), leading to 41.3% of all ewes being scanned for multiples. The mean pregnancy rate differed significantly between scan types (P < 0.001) and was higher in mob scanned for multiples. The mean pregnancy rate in ewes SIL/Empty was 0.72 ± 0.27 and the median pregnancy rate in mobs scanned for SIL/Empty was 0.84. In mobs scanned for multiples, the mean pregnancy rate was higher at 0.84 ± 0.15, while the median pregnancy rate was 0.89.

**Figure 3:**
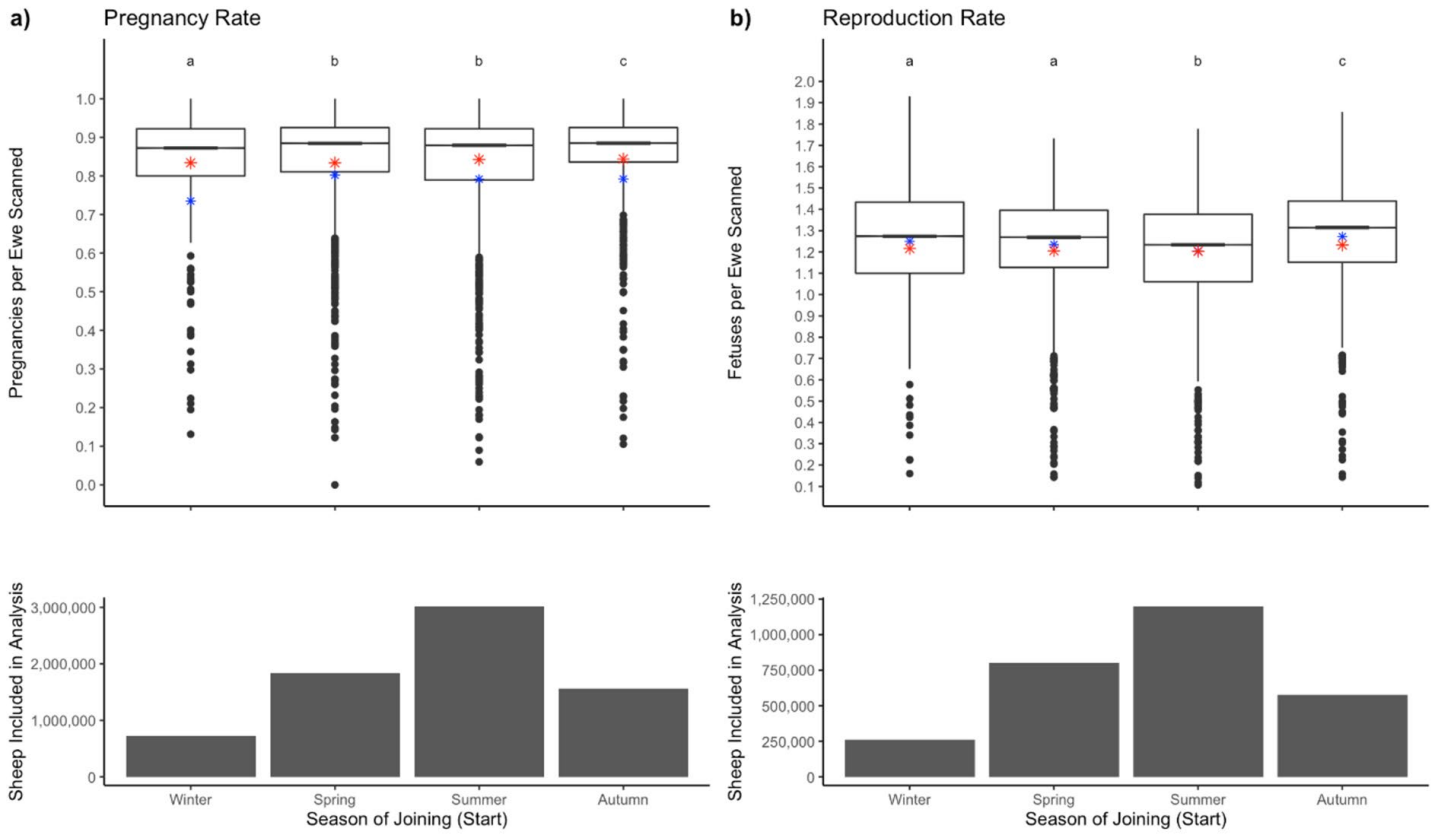
The effects of season on pregnancy scanning results, with column graphs indicating ewe numbers scanned. Panel (**a**) Pregnancy rate (ewes pregnant per ewe scanned) for all ewes scanned. Panel (**b**) Reproduction rate (fetuses per ewe scanned). Boxplots indicate the range, average (red star), median (black bar) and predicted mean (blue star). Superscripts with different letters denote significant differences.

**Figure 4:**
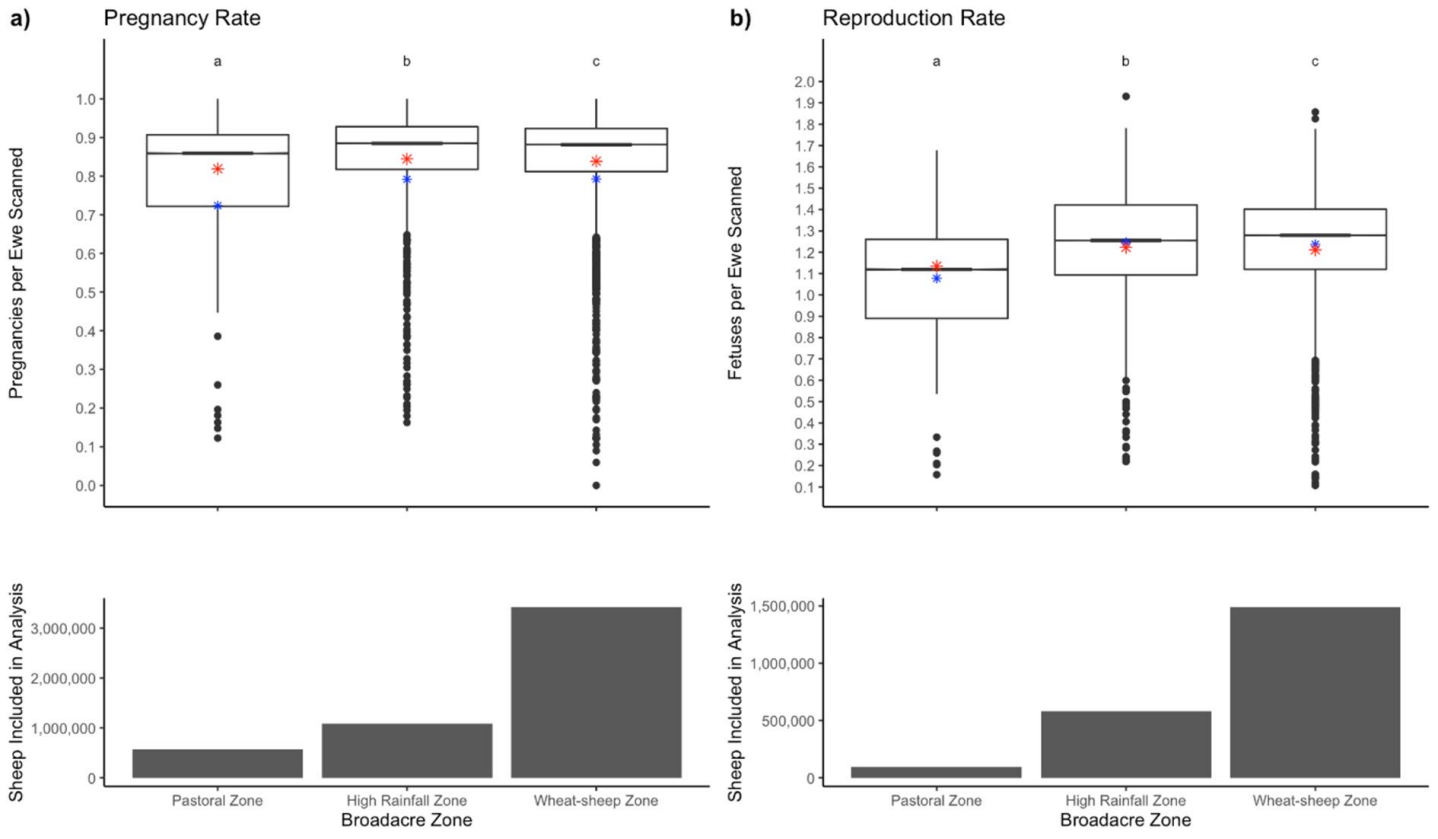
The effect of broadacre zone on scanning results, with column graphs indicating the number of ewes scanned. Panel (**a**) Pregnancy rate (ewes pregnant per ewe scanned) for all ewes scanned. Panel (**b**) Reproduction rate (fetuses per ewe scanned). Boxplots indicate the range, average (red star), median (black bar) and predicted mean (blue star). Superscripts with different letters denote significant differences.

The factors significantly associated with pregnancy rate included season (P < 0.001, Fig. 3) and broadacre zone (P < 0.001, Fig. 4). Mobs mated in spring (P < 0.001), summer (P < 0.001) or autumn (P < 0.001) had higher pregnancy rates than ewes mated in winter. Pregnancy rates were higher when mobs were mated in summer compared to spring, in autumn when compared to summer, and in autumn when compared to spring. Pregnancy rate was higher in flocks mated in the high rainfall zone (P < 0.001) and wheat-sheep zone (P < 0.001), when compared to the pastoral zone, and were higher in the wheat-sheep zone than in the high rainfall zone. Mean pregnancy rate in the Australian pastoral zone was 0.72 ± 0.24, in the sheep-wheat belt was 0.76 ± 0.25 and in the high rainfall zone was 0.76 ± 0.25, with mean reproduction rates of 1.10 ± 0.31, 1.21 ± 0.26 and 1.22 ± 0.27, respectively.

Among mobs that were scanned for multiples, pregnancy rate in mobs mated in spring (P < 0.01) or summer (P < 0.001) were lower than mobs mated in winter, while higher in autumn-mated mobs compared to winter-mated mobs (P < 0.001). Pregnancy rates were lower in summer mated mobs when compared to spring, but higher in autumn-mated mobs when compared to summer and spring. For mobs scanned for multiples, pregnancy rate was higher in those mated in the wheat-sheep zone than in the high rainfall zone, and both zones had higher pregnancy rates than the pastoral zone.

Reproduction rate is only calculated on data where the mobs were scanned for multiples. Spring (P < 0.001) and summer-mated (P < 0.001) mobs had lower twinning rates when compared to winter-mated mobs, while autumn-mated mobs had a higher twinning rate (P < 0.001) than winter-mated mobs. Reproduction rate was also lower in summer mobs compared to spring and higher in autumn mobs when compared to mobs mated in summer or spring. Reproduction rate was higher in mobs mated in the wheat-sheep zone when compared to those mated in either the pastoral or high rainfall zone. High rainfall zone mobs had a higher reproduction rate that the pastoral zone.

### Ewe age

When compared to ewe lambs, maidens (P < 0.001) and mature (P < 0.001) ewes had a higher pregnancy rate, and mature ewes had a higher pregnancy rate than maiden ewes. Subsets of the data for ewe age indicated mean pregnancy rates for lambs, maiden and adults were 0.51 ± 0.32, 0.78 ± 0.24 and 0.79 ± 0.23, with mean reproduction rates of 0.81 ± 0.38, 1.15 ± 0.22 and 1.26 ± 0.24, respectively. When the analysis for pregnancy rate was performed only on mobs scanned for multiples, the same was observed, with higher pregnancy rates in maiden (P < 0.001) and mature (P < 0.001) ewes than ewe lambs, and higher in mature ewes than maidens.

**Figure 5:**
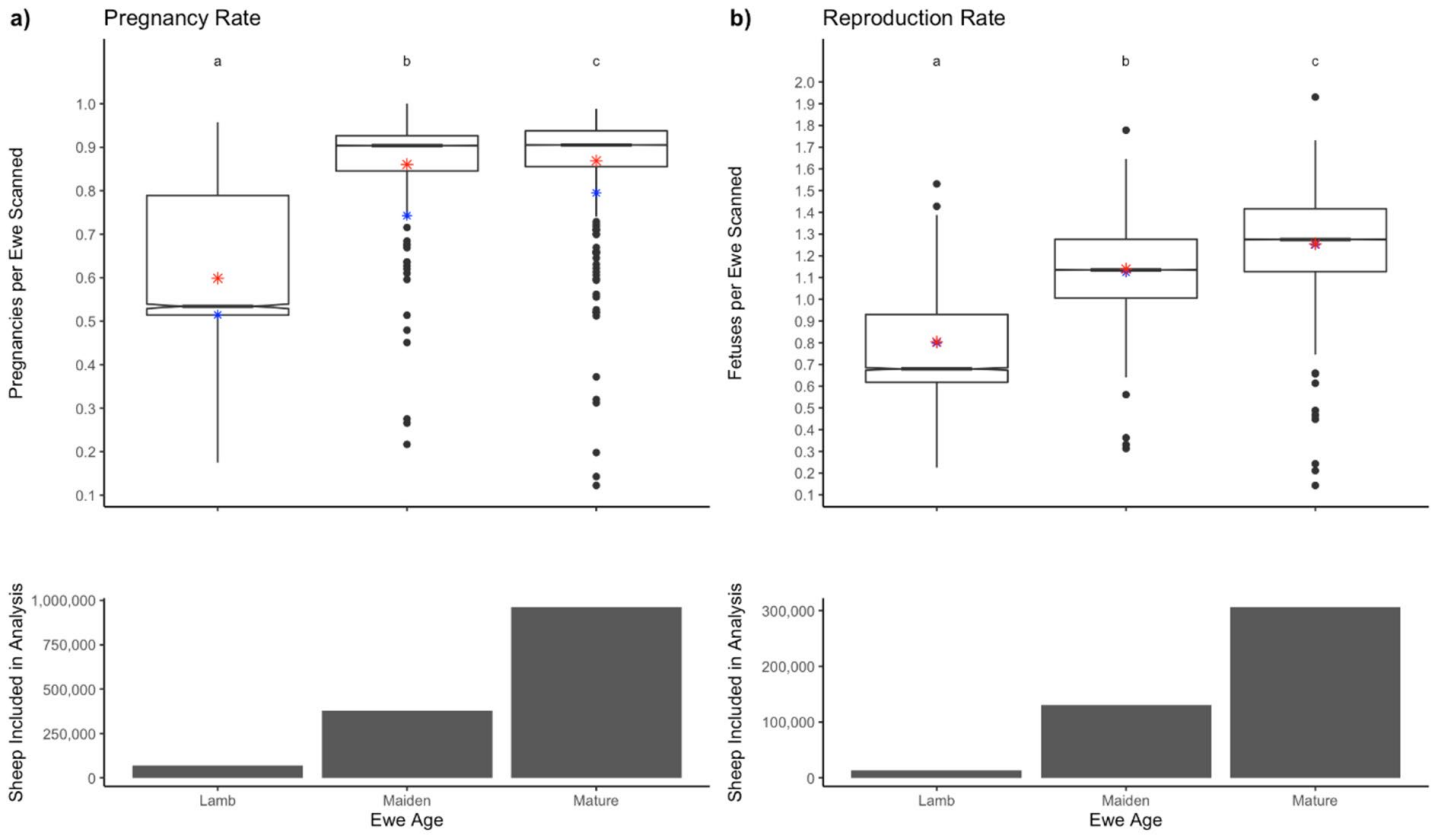
The effects of age on pregnancy scanning results. The column graphs indicate the number of ewes scanned. Panel (**a**) Pregnancy rate (ewes pregnant per ewe scanned) for all ewes scanned. Panel (**b**) Reproduction rate (fetuses per ewe scanned). Boxplots indicate the range, average (red star), median (black bar) and predicted mean (blue star). Superscripts with different letters denote significant differences.

Clear differences in the reproduction rate were apparent between each age class (Fig. 5), which was higher in maiden (P < 0.001) and mature (P < 0.001) ewes than in ewe lambs and was higher in mature ewes than in maidens. Relationships between the seasons of mating in the ewe age subset differed where the pregnancy rate mobs scanned for multiples were higher in summer-mated flocks than autumn- and spring-mated flocks, and spring-mated flocks were higher than autumn-mated flocks (Supplementary Table 1).

### Ewe breed

Shedder ewes had lower pregnancy rate and reproduction rates when compared to all other ewe breeds (Table 2, P < 0.001). The pregnancy rate of maternal ewes was higher than terminal ewes, while highest in Merino ewes. When examining the pregnancy rate of multiple-scanned flocks only, the same relationships were observed with all breeds recording higher pregnancy rates than Shedder mobs (P < 0.001). Similarly, in multiple-scanned mobs, the pregnancy rate of maternal ewes was higher than terminal ewes, while highest in Merino ewes. The subset for breed indicated mean pregnancy rates for hair/shedding sheep, terminal, maternal and Merino breeds of 0.69 ± 0.23, 0.78 ± 0.21, 0.79 ± 0.23 and 0.80 ± 0.21, and mean reproduction rates of 1.02 ± 0.31, 1.22 ± 0.26, 1.22 ± 0.27 and 1.24 ± 0.22, respectively.

**Table 2:** Exponentiated beta coefficients for pregnancy rate of all mobs scanned within subsets for breed, mob size and method of joining, including pairwise comparisons.

Model	Factor	Beta coefficient	Difference in beta coefficients	Exponentiated beta-coefficient	No. of ewes	Pregnancy rate (Ewes pregnant per ewe scanned)	Reproduction rate (Fetuses scanned per ewe scanned)
Breed	Shedders (intercept)	0.09			75,372	0.687	1.02
	Terminal	0.66	− 0.57	1.446	375,088	0.782	1.23
	Maternal	0.62	− 0.53	1.410	29,417	0.785	1.22
	Merino	0.68	− 0.59	1.465	1,269,739	0.802	1.24
	Maternal : Terminal		0.040	1.293			
	Merino : Terminal		− 0.020	1.332			
	Merino : Maternal		− 0.060	1.341			
Mob size	< 100 ewes (intercept)	0.03			98,442	0.633	1.05
	100–200	0.4	− 0.37	1.242	461,075	0.731	1.15
	200–500	0.63	− 0.60	1.432	2,027,809	0.789	1.23
	500–1000	0.61	− 0.58	1.414	2,002,190	0.786	1.24
	> 1000	0.7	− 0.67	1.499	2,853,798	0.789	1.22
	200–500 : 100–200		− 0.230	1.352			
	500–1000 : 100–200		− 0.210	1.337			
	> 1000 : 100–200		− 0.300	1.407			
	500–1000 : 200–500		− 0.020	1.318			
	> 1000 : 200–500		0.070	1.261			
	> 1000 : 500–1000		− 0.090	1.357			
Method of joining	Embryo transfer (intercept)	0.11			3,023	0.638	0.89
	Artificial insemination	0.17	− 0.06	1.088	59,867	0.719	1.08
	Natural	0.53	− 0.42	1.330	7,380,108	0.762	1.21
	Natural : Artificial insemination		− 0.360	1.320			

Among multiple scanned mobs, all ewe breeds had a higher reproduction rate than the shedder mobs (P < 0.001), while maternal ewes had a higher reproduction rate than terminal ewes, which was highest in Merino ewes. Within the ewe breed subset, pregnancy rate was higher in spring than summer and autumn, and higher in summer than autumn.

### Mob size

Pregnancy rate varied between mobs of different sizes, being the lowest in mobs < 100 ewes when compared to all other mob sizes (P < 0 0.001, Table 2). For mobs that scanned for multiples, higher pregnancy rates were observed as mob size increased from < 100 ewes (P < 0.001). Reproduction rate followed the same pattern, as mobs with < 100 ewes found to have the lowest reproduction rate (P < 0.001). As mob size increased to > 1000 ewes, reproduction rate continued to increase.

### Method of joining

Table 2 shows that pregnancy rate in mobs that were embryo transfer recipients was lower than artificially inseminated (P < 0.01) or naturally mated mobs (P < 0.001). Among multiple scanned mobs, pregnancy rate was higher in mobs that were artificially inseminated (P < 0.01) and naturally mated (P < 0.001) when compared to the embryo transfer recipient mobs and was higher in naturally mated mobs than the artificially inseminated mobs.

The reproduction rate of artificially inseminated mobs (P < 0.001) and naturally mated mobs (P < 0.001) was higher than embryo transfer recipient mobs and was higher in naturally mated mobs when compared to the artificially inseminated mobs.

### Variation over time

**Figure 6:**
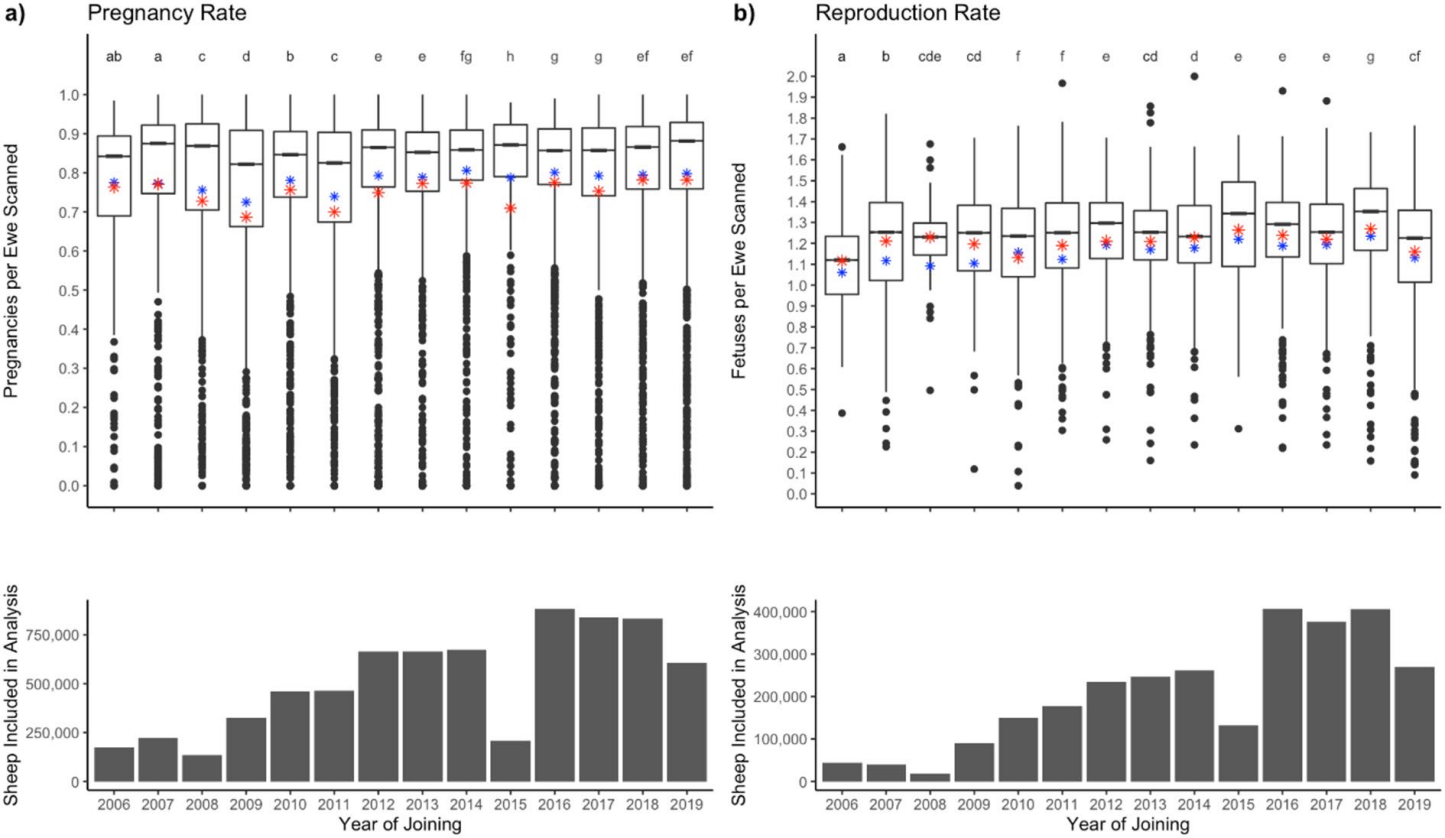
The effects of year on pregnancy scanning results, with column graphs indicating ewe numbers scanned. Pregnancy rate (ewes pregnant per ewe scanned) (Panel (**a**) and Reproduction rate (fetuses per ewe scanned; Panel (**b**) reported for each year. Boxplots indicate the range, average (red star), median (black bar) and predicted mean (blue star). Superscripts with different letters denote significant differences.

The effect of year was marginal, with the explanatory power of the model being weak with a conditional R^2^ of 0.02 and a marginal R^2^ of 0.009 (Fig. 6). Small improvements in pregnancy rate appear to be denoted in the superscripts of Fig. 6, however, that did not translate to consistent increases in the reproduction rate.

### The correlation between pregnancy rate and reproduction rate

**Figure 7:**
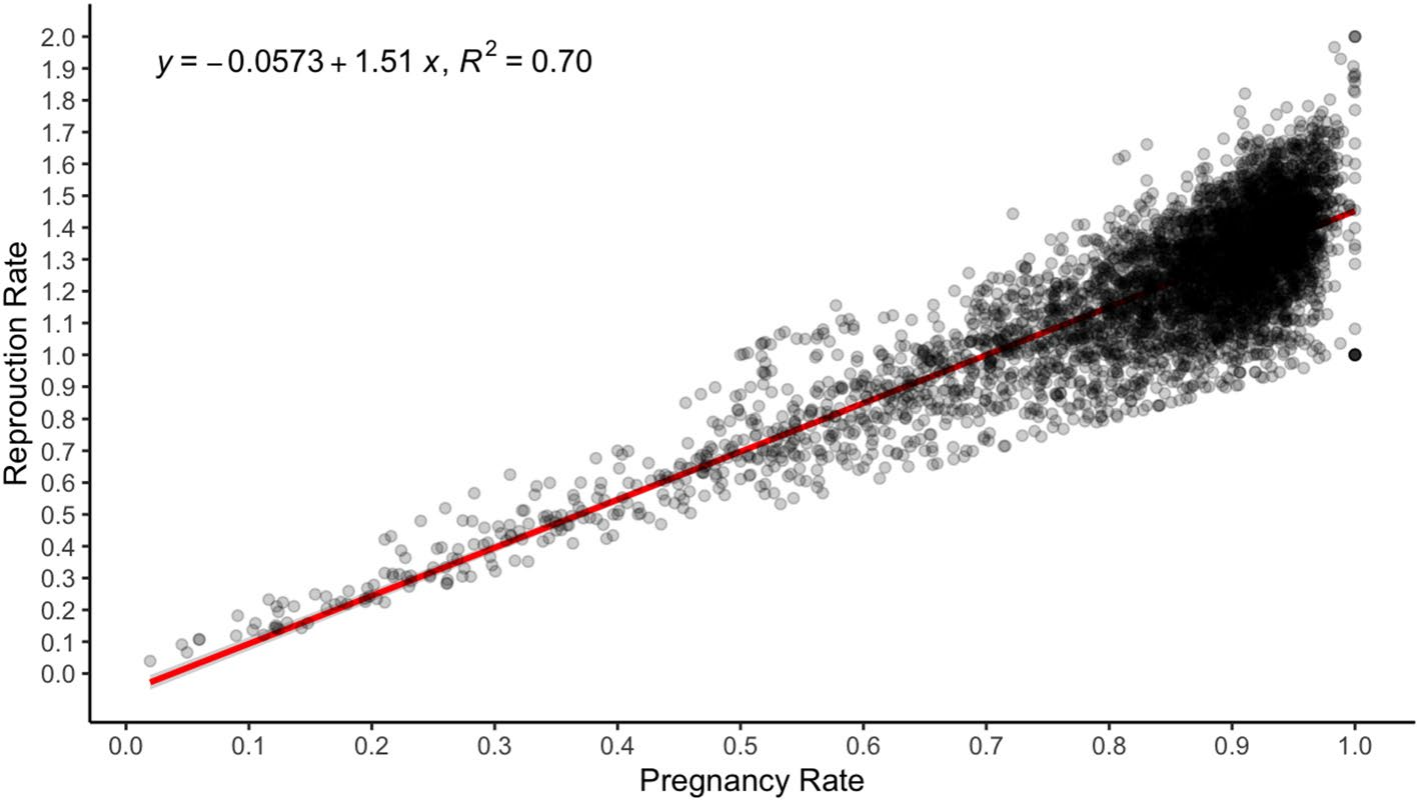
The correlated relationship between reproduction rate and pregnancy rate, including the linear trend (red line).

Using data limited to mobs scanned for multiples, a correlation was performed plotting pregnancy rate and reproduction rate, showing a positive relationship (y = −0.0573 + 1.51x), where x = pregnancy rate and y = reproduction rate. The coefficient of determination was 0.70. The regression formula implies a mean litter size of 1.45 fetuses per pregnant ewe (Fig. 7) in ewes scanned for multiples.

## Discussion

This paper presents the results of a large Australian sheep pregnancy scanning dataset and the compelling finding suggests the average Australian sheep flock that is pregnancy scanned has a fertility problem. The mean *per ewe* fertility for mobs scanned for pregnancy status only was 0.76 ± 0.24, which falls well below the 87% observed in another pregnancy scanning cross-sectional study^20^. The overall median pregnancy rate was a better result than the average but remained low at about 0.83 in SIL mobs. The mean pregnancy rate for multiple-scanned mobs was higher at 0.84 ± 0.15, for which the mean reproduction rate was 1.21 ± 0.27, suggesting sheep flocks that are ultrasound pregnancy scanned for multiple litters are likely provided with improved nutritional management and/or selection for reproduction and freer of disease and stressors.

Using the linear regression created in the present study, estimates for the mean reproduction rate for the average mob with a pregnancy rate of 0.76 ± 0.24 equates to a mean of 1.09 ± 0.31 fetuses per ewe scanned. The median pregnancy rate (0.83) equates to a reproduction rate of 1.20 and is closer to expectation. For example, the next largest Australian pregnancy scanning dataset reports a mean reproduction rate of 129%^21^. Using our linear regression, a reproduction rate of 129% has a pregnancy rate of 89%, however, that particular data was supplied by sheep producers in Western Australia and may not be a true reflection of all flocks in that state or the national flock.

To reconcile the low pregnancy rate observed in the present study, we refer to publications reporting results for large studies. In a review of potential for pregnancy scanning data to be included in genetic evaluation programs, Bunter, et al.^22^ reported on the outcomes of over 46,600 ewes and observed pregnancy rates between 80 and 86%. Using over 110,000 records from multiples genetics research flocks, Safari, et al.^23^ reported 80% of ewes lambed per ewe joined, with 135% lambs born per ewe lambing, which is a reproduction rate of 108%. It is likely, however, that pregnancy rates from genetics research studies will have employed single-sire mating systems or artificial insemination, which may explain generally why such flocks had low pregnancy rates.

Few large-scale publications focusing on commercial ewe pregnancy rates are available for comparison. Fowler^20^ reported a pregnancy rate of 86.3% for 98,272 ewes, Bates, et al.^24^ found a 90% pregnancy rate for 29,157 mature ewes in a multi-breed survey, and in assessing 34,645 ewes, Kilgour^25^ found a pregnancy rate of 94–95%. None of these studies reported mean pregnancy rates as low as the present study.

More large-scale publications are available for reproduction rate than are for pregnancy rate. The findings of which varied from 110%^20^, 123%^25^, 126–132%^26^ and 140%^24^. From these studies, the present study sits within the range observed.

In a survey of Victorian farms, Armstrong, et al.^10^ found variation between breeds and seasons of mating for reproduction rate, ranging between 116 and 144%, similar to the findings of the present study. Across each of these aforementioned studies, pregnancy rates and reproduction rates vary considerably. The present study has revealed an unexpected outcome that requires a more detailed investigation into the fertility of the Australian breeding ewe because of the sheer size of the dataset and its duration over time.

The proportion of mobs scanned for multiples was 31% of all mobs scanned, which is half of the proportion of producers claiming to pregnancy scan for multiples, according to the findings of a producer-practice survey, reported by Howard and Beattie^27^. However, in the present study the average mob size was larger in mobs scanned for multiples, where 41% of the total number of ewes scanned had litter size determined.

Kleemann, et al.^28^ reported a regression formula between body condition score (BCS; 1 to 5 score range, where 1 is lean and 5 is obese) and fertility in adult ewes (y = 12.54x + 48.97) for South Australian sheep flocks, which when applied to the findings of the present study, we speculate, suggests the mean pregnancy rate (y) was achieved by ewes with a body condition score (x) around BCS 2, whereas the ewes scanned for multiples were closer to BCS 2.75. This implies the medium-term pre-mating nutritional state of the average Australian ewe tends toward undernourishment, but this isn’t the only factor affecting fertility.

The role that previous reproduction has on current reproduction may be a factor impacting fertility. Kleemann, et al.^29^ observed that 50% of ewes scanned empty as a two-year-old ewe will be empty as a three-year-old, and 43% of empty three-year-old ewes will be empty as a four-year-old. Such detail is not available in mob-based data sets but cannot be discounted as an effect on the present results but may contribute to the number of empty ewes if culling pressure is decreased as a result of a flock rebuilding phase, or if empty ewes are not identified accurately to enable the culling of twice-empty ewes. However, the 14 year timescale of the present study will account for the temporal effects of retaining low fertility ewes. In some situations, empty ewes will be remated, leading to smaller mob sizes present for pregnancy scanning that have a tendency for lower pregnancy rates. This may help explain why smaller sized mobs had the lower pregnancy rates.

Age has a clear impact on young ewe pregnancy scanning outcomes. From a genetics study, Afolayan, et al.^30^ reported pregnancy rates in ewe lambs of 49% to 63% and 80% to 82% in hogget ewes. Elsewhere, seedstock yearling ewes that were naturally mated had an average pregnancy rate of 54% and a reproduction rate of 76%^31^. In the present study, ewe lamb pregnancy rate was higher on average than reported in Bunter but had a similar reproduction rate.

Differences in pregnancy rate between types of mating were in line with expectation, where mobs mated by embryo transfer or artificial insemination were lower than naturally mated flocks. The pregnancy rates in artificially inseminated ewes averaged 72% across Merino and first cross ewe breeds^32^, which is similar to the present study, while the reproduction rates were much higher than observed in the present study.

The information provided is real world data, but the reasoning behind the farm management decision to pregnancy scan the sheep cannot be explained by the available information. In mobs where multiples were identified, a higher pregnancy rate was observed, suggesting that producers pursuing higher lamb weaning rates are managing ewe and ram preparation better in the peri-mating period. When contrasted to the pregnancy rates in published literature, the present result is surprisingly low and difficult to explain. We propose four possible scenarios in an attempt to understand the nature of the data.

Scenario 1. It is possible that commercial sheep producers willing to take-part in active R&D may be higher performing sheep producers, sufficiently confident in their own sheep and management to allow researchers onto their properties to record information. If true, this scenario suggests the literature (our understanding of pregnancy rates) could be biased by better sheep managers and flocks with better reproduction.

Scenario 2. It is possible that the commercial sheep producers contracting the pregnancy scanning businesses sourced for this study have done so because of a belief there was a temporal issue with the pregnancy rates in their flocks. If this scenario is true, then there is a problem with Australian sheep fertility in the average flock, but which may bias the results of the database, and in particular the SIL/Empty scanned mobs.

Scenario 3. It is also possible, and most likely, that Australian sheep producers engage pregnancy scanning services not because they suspect a problem with fertility or litter size, but to find pregnant and non-pregnant ewes. If this scenario is true, then there is a problem with Australian sheep fertility.

Scenario 4. It is possible that the pregnancy scanning businesses supplying these data are servicing clients with fertility rates that are lower than the rest of the Australian sheep flock because of historically low reproduction rates. If this scenario were true, then the data is biased.

Elsewhere, the producer-supplied pregnancy scanning dataset for the Western Australian sheep producers^21^ suggests a mean reproduction rate of 129%, using data supplied for 684 flocks with 1,277,339 ewes, at the time of writing. Pregnancy rate was not supplied by the producers for that dataset. It is possible that the western Australian dataset may also be biased by those producers willing to engage and share data. Furthermore, that dataset is limited to those scanning for multiples. Nevertheless, the mean reproduction rate is not too dissimilar to the result in the present study implying the data of the present study is a good reflection of the real world.

The number of pregnancy scanners in Australia exceeds 160 operators^33^ that may be assessing the sheep of 50% of Australian producers^27^. There are approximately 42.5 M ewes in Australia^34^, which implies that around 21 M ewes are pregnancy scanned annually. Over an equivalent timeframe of 14 years, there may well have been 294 M ewes scanned in Australia, suggesting the scale of the present dataset, while large in comparison to all other published reports, is only 2.5% of the population. Thus, ours is a sample upon which we make inferences about the Australian sheep flock’s fertility. Therefore, a large amount of sheep reproduction information is being created and technically available, but little of it is being used, except for the interaction between scanner and client. If collected, these data would provide more accurate information on variability, and thus may better inform strategic research and development investments as well as extension and adoption activities, but it is clear that annotation of the mob-based records is necessary for more detailed understanding of the factors affecting the Australian ewe flock.

### Supplementary Information

Supplementary Table1.

## Data Availability

The datasets generated during and/or analysed during the current study are not publicly available due to the data being commercially sensitive, however a modified dataset will be available from the corresponding author on reasonable request.
